# How Shall We Train Our Boys?

**Published:** 1878-07

**Authors:** 


### HOW SHALL WE TRAIN OUR BOYS?

BY UNCLE BOB.

As oft repeated as the ever recurring generation is the query of "our boy, what shall we do with him?" We know well enough what to do with our neighbor's boy, and we wonder that they cannot see as plainly as we. Still, we cannot go to them and point out the defects in their system of training. As I look back to the days when we boys began, it seems as though the average boy then was somehow quite different from the one now. He did not leave undone ninety and nine things he was charged to do, and per contra do everything he ought not to. He was not out of hearing when called, and could come somewhere within a reasonable time when sent for. He had some show of respect for the age of those about him, and could find time to say *Mr.* Jones' house as easily as Jones' house. He did not call his neighbor Brown, "Old Brown," because he was simply not popular with him. 'Twas not necessary to have springs on every door in the house to ensure having them latched when he passed through. A clothes hook was understood to be intended to hang something upon, instead of designating the place to throw something under. Cats could come in and out of the house and keep their temper in his presence. Rainy weather did not always make him so sour that he could not remain indoors while the floods descended. He could find time to run of an errand if asked, and could recollect whether he was sent to take, instead of bring, an article from the neighbors. He did not consider every one cross, who, being a few years older than he, interfered with his plans. In fact, he was not a make up of only negative bad qualities. He had, as I recollect him, some positive good points, for doing or not doing which, he could be praised. He gave promise of *being something* before he was in his teens.

Is it an idea, or a fact, that boys now are much worse than then? What has made this change in the appreciation we have for our boys. I think Uncle Bob would get no chance to say another word if he sat listening to the reproaches put upon the boys. No doubt many would be just, for boys of one generation *are not* like the ones preceding them in some respects. The "fastness" of the present age has and should leave a mark upon all. Whether there is to remain a permanence of unlovely characteristics, is the fault of us who bring up our boys. I suspect that *our* fathers and mothers felt much as we do now, and that they, too, wondered what would become of *us*. Let us remember that we have grown older, and not the boys younger—that our eyes take in the natural objects of sight with a varying vision as age comes upon us—that we have, perhaps, *grown away* from the personalities of boyhood times. Appreciation of youth is a failing faculty with most every one, hence boys don't pass for what they are—are condemned sometimes unjustly, by reasons of our wrong inferences, or an unwillingness to think of them as we should.

Perhaps we forget how much patience our parents exercised toward us; forget how skillful they were to encourage as well as to repress their youngsters; forget how they sought to develope *good* characteristics and conquered in us many an evil propensity. Alas! they are dead and gone, and Elijah's mantle has not fallen upon us, enabling us to go on in their good work.

I suppose I migh enumerate by the dozen, reasons, or suggestions, at least, for the comparative difference in the behavior of the past and present generation, for I am ready to admit that there is certainly a difference in the outward show of character and disposition of some families.

The most prominent, and the one I shall speak of *as* a prominent reason, is the want of persistence in training up our boys. Whenever we say to them that they must or they must not do this or that thing, we must follow up the injunction and know that our word has availed as intended. The benefit we seek is lost if we do not know that we have enforced obedience and with it, also, respect. To punish sometimes and overlook at others, will result in making our children doubtful whether we really *care* if we are implicitly obeyed or not. The true benefit of all punishment is the certainty that it will come. If we knew that sometimes red hot iron would burn us and sometimes not, or that sinking over our heads in the water would or would not drown us, according to the mere whim of the Creator, we should constantly try the chances according as our inclination led us to try the carrying out of a purpose which involved the peril. To promise John a whipping for an act, and then fail to know whether he had been guilty of disobeying, puts a premium upon his sagacity in blinding us as to his conduct. It is of no use to go about promising and never fulfilling. With a score of laws that have an uncertain penalty, a community invites lawlessness.

To the question, would you punish in anger, I would say *yes*, rather than no punishment should ensue. There is more benefit from such punishment, if it causes offenses to stop and be considered, than an indefinite, hesitating, loving kindness that is never around when danger has threatened. A certain physician was asked once if he would justify the hanging of a criminal who was sentenced to death and great doubts existed as to the grounds of a plea of insanity. Said he, "Yes, I think that he certainly would never kill another man. Society would be safer at any rate." So I say society would be safer for the repression of that boy's evil intents when our indignation was aroused, than if he escaped through uncertain ideas as to whether the violated law would reach him, while we were waiting to cool down. There is nothing to hinder an exhibition of love and care for our children at a thousand times, and in as many ways, and certainly it is not right to make our commands vague from lack of certainty whether we are angry or not. Let our yea be *yea*, and our nay be *nay*, in a signification not given in the usual quotation of the phrase. Upon this I rest a great share of the usefulness of family government. Exercising it, we shall know far better how to solve the query of "our boys." I close with the injunction to be persistent in enforcing every law of the family, that there may be no uncertainty as to the intent for which it was given, thereby rendering our children mindful of the fact that there are *consequences* to follow violations.

